# Formation and Stability of Smooth Thin Films with Soft Microgels Made of Poly(*N*-Isopropylacrylamide) and Poly(Acrylic Acid)

**DOI:** 10.3390/polym12112638

**Published:** 2020-11-10

**Authors:** Elena Buratti, Ilaria Sanzari, Franco Dinelli, Themistoklis Prodromakis, Monica Bertoldo

**Affiliations:** 1 Istituto per i Processi Chimico Fisici del Consiglio Nazionale delle Ricerche (IPCF-CNR), sede di Pisa, via Moruzzi 1, 56124 Pisa, Italy; elena.buratti@roma1.infn.it; 2 Istituto dei Sistemi Complessi del Consiglio Nazionale delle Ricerche (ISC-CNR), sede Sapienza, Pz.le Aldo Moro 5, 00185 Roma, Italy; 3 Zepler Institute for Photonics and Nanoelectronics, Highfield Campus, University of Southampton, Southampton SO17 1BJ, UK; is1v14@soton.ac.uk (I.S.); T.Prodromakis@soton.ac.uk (T.P.); 4 Istituto Nazionale di Ottica del Consiglio Nazionale delle Ricerche (INO-CNR), via Moruzzi 1, 56124 Pisa, Italy; franco.dinelli@ino.it; 5 Istituto per la Sintesi Organica e la Fotoreattivitá del Consiglio Nazionale delle Ricerche (ISOF-CNR), via P. Gobetti 101, 40129 Bologna, Italy; 6 Dipartimento di Scienze Chimiche e Farmaceutiche, Università degli Studi di Ferrara, via L. Borsari, 45121 Ferrara, Italy

**Keywords:** microgels, PNIPAm, PAAc, IPN, thin films, spin-coating

## Abstract

In this work, soft microgels of Poly(*N*-Isopropylacrylamide) (PNIPAm) at two different sizes and of interpenetrated polymer network (IPN) composed of PNIPAm and Poly(Acrylic Acid) (PAAc) were synthesized. Then, solutions of these different types of microgels have been spin-coated on glass substrates with different degrees of hydrophobicity. PNIPAm particles with a larger diameter form either patches or a continuous layer, where individual particles are still distinct, depending on the dispersion concentration and spin speed. On the other, PNIPAm particles with a smaller diameter and IPN particles form a continuous and smooth film, with a thickness depending on the dispersion concentration and spin-speed. The difference in morphology observed can be explained if one considers that the microgels may behave as colloidal particles or macromolecules, depending on their size and composition. Additionally, the microgel size and composition can also affect the stability of the depositions when rinsed in water. In particular, we find that the smooth and continuous films show a stimuli-dependent stability on parameters such as temperature and pH, while large particle layers are stable under any condition except on hydrophilic glass by washing at 50 °C.

## 1. Introduction

Stimuli-responsive thin films have received a great deal of interest in recent years because they are capable of altering their chemical and/or physical properties upon exposure to variations in temperature, pH, salt concentration, light, electric, or magnetic field [1,2]. They are increasingly employed for the creation of smart systems for applications in a variety of different fields, such as in drug delivery [3,4], biomedical applications [5,6], robotics [7], and sensors [8,9]. They can be seen as a sort of smart building blocks for more complex engineered materials [10,11,12,13], such as for the fabrication of miniaturized devices with fast response time, exploitable in coatings [14], bio-interfaces and bio-separation [15], micro- and nano- actuators [16], sensors [12,17,18,19], and tissue engineering [20,21].

Poly(*N*-isoproprylacrylamide) (PNIPAm) is one of the most used thermo-responsive polymers. It is especially used in biomedical application area, having a low critical solution temperature (LCST) around 32 °C, which is close to the human body temperature. Below this value, the polymer chains are hydrophilic and thus hydrated when dispersed in water; above this value, they become hydrophobic and insoluble in water, leading to a sharp but reversible coil-to-globule transition. This property has been exploited to prepare surfaces that can change their polarity through control of the external temperature [13,19,22,23,24].

Responsive films can be prepared using different techniques. Some classic methods are radiation-induced graft polymerization [25,26], surface-initiated living radical polymerization (“grafting from”) [27,28], or grafting of preformed polymer chains to the surface (“grafting to”) [29,30]. These procedures allow one to obtain brushes or crosslinked hydrogels but present some drawbacks—e.g., the need for expensive equipment, a low grafting degree, chemical residual from the synthesis, and sometimes a poor control on the film thickness [20,31]. In recent years, microgel-made films have been proposed as an alternative to in-situ formed hydrogels, because they can be easily prepared, their composition can be more easily tuned and often they offer better mechanical properties than analogues produced with traditional methods [22,32,33,34,35].

Microgels are colloidal particles, made by a three-dimensional crosslinked network dispersed in a suitable solvent. If made of responsive soft materials, they can change their size and shape, compressing or even interpenetrating each other upon the application of external stimuli. Microgels with a variety of composition and architecture with corresponding rational design properties, including multi-responsiveness, are able to meet increasingly complex demand [36] and can be obtained thanks to the recent advances in colloidal chemistry [37]. Even more advantages can be found in using microgel for thin film preparation, as for example no residual monomers are present in the final product, since the microgel dispersion is synthesized and purified before deposition, making it appealing for biomedical applications [38].

PNIPAm microgels were firstly synthesized by Pelton [39] using a precipitation polymerization, a method nowadays already optimized. By adding a second component to the PNIPAM network, multi-responsive microgels have been also obtained. In particular, acrylic acid (AAc), a pH-responsive repeating unit, has been introduced as co-monomer to control the volume-phase-transition (VPT) of microgels through pH [40,41,42], ionic strength [43], or electric field [44]. This is very promising especially for soft robotics [45].

The responsiveness of multicomponent systems usually depends on the relative content of the individual components as well as on their architecture. Accordingly, in the case of random copolymers of NIPAM and AAc, P(NIPAM-*co*-AAc), the response temperature can be controlled trough the AAc content [46]. In the case of physically interpenetrated polymer network (IPN), made of PNIPAM and Poly(Acrylic Acid) (PAAc), the response temperature (LCST) of the system is not affected by the presence of PAAc. However, the system exhibits additional properties being able to switch its solubility, and ionic charge with the pH value [47,48,49]. Furthermore, the amount of incorporated PAAc network affects the density as well as the softness and thus, the mechanical properties of the system [46,50].

Microgels films have been obtained by different deposition methods such as dip-coating, spin-coating, solvent evaporation, or layer-by-layer deposition [17,22,38,51,52,53,54,55,56,57,58,59,60]. Despite the numerous advantages of these strategies, some drawbacks yet exist: (a) scarce stability when immersed in water; (b) low packing density. The stability issue can be overcome by multiple ionic interactions or covalent bond formation between microgels and substrate [51,58,59,61,62]. This aspect is particularly important, for instance, during cell growth by immersion in buffers or complex aqueous solutions at 37 °C to avoid cell detachment from the substrate [63].

On the other hand, a low pack density usually results in discontinuous islands of particles or in bumped morphology films due to the sphere arrangement [51,52,53,54,58,60,64]. Only occasionally continuous and smooth films consisting of microgels, merged to a certain degree, have been reported [22,60] and even in these cases the particle edges are easily detectable. However, PNIPAm particles with a diameter in the sub-micrometric range (<100 nm) have been recently reported to behave more like polymeric coil rather than rigid spherical particles, even if crosslinkers are used in the synthesis [41,65]. Accordingly, we have then produced a very smooth film morphology, comparable to the one obtained with linear polymers, by spin-coating PNIPAm microgels with a diameter of 70 ± 5 nm [38].

In order to further investigate that observation, in this work we have studied the effects of the particle size on thin film morphology and stability. In particular, the reduced size allows one to control the film interaction with the substrate through temperature, enabling the hydrophobic/hydrophilic transition, and trigger the film stability. Glass surfaces with different degrees of hydrophobicity have been prepared by hydrolysis with NaOH, by silanization with methacrylic moieties of intermediate polarity and by silanization with high hydrophobic octyl chains. The effects of surface hydrophobicity on the film formation and stability have been evaluated. Moreover, we describe the effects of the insertion of polar AAc functionalities on both film morphology and stability. PAAc, inserted with PNIPAm in an IPN architecture, introduces the possibility to control the microgel interaction with the substrate through pH. In fact, the presence of ionizable groups, such as carboxylic acid of AAc, has been reported to affect both the packing density of the particles and their interaction with the substrate [58].

## 2. Materials and Methods

### 2.1. Materials

*N*-isopropylacrylamide (NIPAM) (Sigma-Aldrich, St. Louis, MO, USA), purity 97%, and *N*,*N*′-methylene-bis-acrylamide (BIS) (Eastman Kodak, Rochester, NY, USA), electrophoresis grade, were purified by recrystallization from hexane and methanol, respectively, dried under reduced pressure (0.01 mmHg) at room temperature and stored at −20 °C. Sodium dodecyl sulphate (SDS), purity 98% and potassium persulfate (KPS), purity 98% were purchased from Sigma-Aldrich (St. Louis, MO, USA) and used as received. Ultrapure water (resistivity: 18.2 MW/cm at room temperature) was obtained with Millipore Direct-Q^®^ 3 UV purification system (Darmstadt, Germany). 3-(trimethoxysilyl)propyl methacrylate (TMSPMA) and trimethoxy(octyl)silane (TMOS) were purchased from Sigma-Aldrich. All the other solvents (Sigma Aldrich RP grade) were used as received. Dialysis membrane, SpectraPor^®^ 1, MWCO 6–8 kDa (Spectrum Laboratories, Inc., Piscataway, NJ, USA) was soaked in distilled water for 2 h and then thoroughly rinsed before use. Cover glasses, round with a diameter of 12 mm, were purchased from Heinz Herenz Medizinalbedarf GmbH (Hamburg, Germany) and used as support for the film deposition.

### 2.2. PNIPAm Microgel Synthesis

Two PNIPAm microgel samples were prepared under standard precipitation method conditions [39]. For sample PNIPAm *a*, 24.162 g (0.214 mol) of NIPAM, 0.4480 g of BIS (BIS/NIPAM molar ratio = 0.013) and 3.519 g of SDS (SDS/NIPAM molar ratio = 5.71) were solubilized in 1550 mL of ultrapure water. The solution was deoxygenated by bubbling nitrogen for 1 h into a 2 L jacket reactor and then heated at 70 ± 1 °C. 1.0376 g (3.84 mmol) of KPS was dissolved in 10 mL of deoxygenated water and added to initiate the polymerization; then the reaction was left to proceed for 16 h. The final dispersion was purified by dialysis (MWCO 6–8 kDa) with distilled water with frequent water change for 2 weeks, then it was concentrated up to 1 and 3 wt% concentration by lyophilization. PNIPAm *b* was prepared as PNIPAm *a* but reducing the SDS/monomer ratio to 3.15 mg per g of NIPAM (SDS/NIPAM molar ratio = 0.12) in the feed of the polymerization.

### 2.3. IPN Microgel Synthesis

For this synthesis, 140.08 g of PNIPAm *a* dispersion at 1.06 wt%, 5 mL (0.0694 mol) of AAc (AAc/PNIPAM mol/g = 0.0467) and 1.1081 g of BIS (BIS/AAc molar ratio = 0.10) were mixed into a 2 L jacketed reactor, diluted with 1260 mL of ultrapure water, deoxygenated by bubbling nitrogen inside for 1 h while kept at 21 ± 1 °C. Then 0.56 mL (3.73 mmol) of TEMED and 0.4441 g (1.95 mmol) of APS were added to start the polymerization and left to proceed for 4 h and 30 min. The sample was purified by dialysis (MWCO 14 kDa) with distilled water with frequent water changes for 2 weeks, iced and lyophilized up to 1 and 3 wt% concentration. As prepared, the obtained dispersions have a pH value of 5.5.

### 2.4. Glass Surface Functionalization

**Scheme 1:**
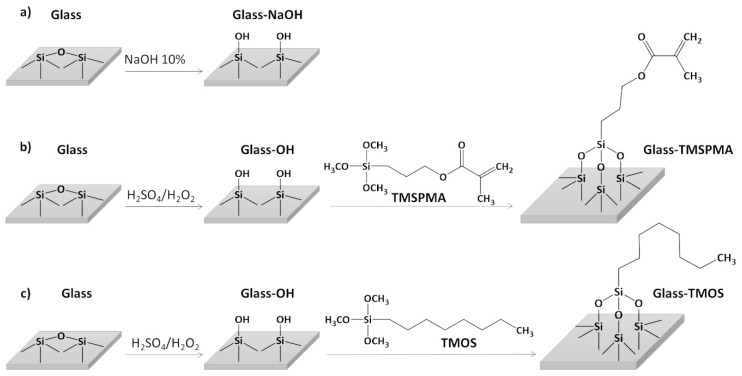
Schematic representation of the procedures employed in order to obtain (**a**) glass-NaOH, (**b**) glass-TMSPMA, and (**c**) glass-TMOS surfaces.

Before any treatment, the cover glasses were rinsed with acetone, isopropanol and distilled water and dried with nitrogen flow. Glass-OH surfaces were obtained by treating the cover glasses for 5 min in a hot 10 wt% NaOH solution while sonicating, followed by extensive washing with fresh water. The procedure was repeated twice and the cover glasses were stored in ultrapure water until use (Scheme 1a). Glass-TMSPMA and glass-TMOS surfaces were obtained by treating the cover glasses with a freshly prepared “piranha” solution (mixture 3:1 of 96% sulfuric acid and of 30 vol% hydrogen peroxide) at 120 °C for 5 min. The cover glasses were then extensively rinsed with distilled water and treated with an acid TMSPMA or TMOS solution (100 mL deionized water, 10 µL acetic acid and 2 wt% TMSPMA or TMOS) overnight at 70 °C, respectively (Scheme 1b,c). The treated surfaces were finally washed with EtOH and dried with a nitrogen flow.

### 2.5. Film Preparation

Spin-coating was done with a POLOS Spin150i/200i infinite apparatus (SPS-Europe, Putten, The Netherlands). For each sample, we prepared dispersions of two different concentrations: 1 and 3 wt% in water. Initially, each cover glass was covered with 60 µL of solution. Spin-coating was then performed following a two-step process. In the first step speed, acceleration and duration values were set at 500 rpm, 500 rpm/s and 30 s, respectively. In the second step, acceleration and duration values were set at 500 rpm/s and 60 s, while the speed value was set at one of the following values: 2000 rpm (R1), 3000 rpm (R2), 4000 rpm (R3), and 5000 rpm (R4).

### 2.6. Characterization Methods

The dynamic light scattering (DLS) analysis was carried out with a Zetasizer Nano series ZEN1600 (Malvern Panalytical, Worcestershire, UK), equipped with a He-Ne laser at a fixed incident angle of 135°. Each sample was diluted at 0.02 wt% in H_2_O and the measurements were carried out at 20 °C in a polystyrene cuvette. The correlation functions were fitted with non-negative least squares (NNLS) in order to obtain the hydrodynamic diameter of the particles.

The ^1^H-NMR analysis was performed with a Bruker Avance DRX 400 spectrometer (Bruker, Billerica, MA, USA). The lyophilized sample was solubilized in deuterium oxide (D_2_O), 99.9% atom of deuterium, at a concentration of 15 g/L and analyzed at room temperature, accumulating 256 scans for each measurement.

Attenuated total reflectance Fourier-transform infrared spectroscopy (ATR FT-IR) were recorded with a FT/IR-6200 spectrometer (Jasco Inc., Easton, MD, USA), equipped with a VeeMAX III (Pike Technologies, Madison, WI, USA) attenuated total reflectance accessory with a Ge crystal. All the spectra were recorded on the lyophilized samples by accumulating 128 scans in the 4000–700 cm^−1^ spectral range

In the elemental analysis, the carbon and nitrogen contents were determined with a Flash EA1112, ThermoQuest NJelemental analyzer (CE Elantech, Lakewood, NJ, USA) on 1–5 mg of pulverized sample in sealed tin combustion boats at 900 °C. The evolved gases were analysed by gas chromatography on molecular sieves at 90 °C with a thermal conductivity detection system. Data acquisition and the elaboration were done with the Thermo Quest CE Instrument Eager 300 Software version 1.01.

The contact angle in static conditions was measured using a Drop Shape Analysis System (DSA 30 Kruss Co., Hamburg, Germany). A water droplet of 5 µL was gradually engaged onto the surface. A polynomial function was fitted to the two 3-phase sections of the profile in the region of the baseline of the droplet [66]. The surfaces were blown with dry nitrogen before each measurement.

The film topographies were recorded in dry conditions by means of AFM (Multimode Nanoscope V, Veeco Instrument Inc., Plainview, NY, USA), working in tapping mode. The cantilevers employed were Al-coated Tap300 Al-G Si tips (Budget Sensors, Sofia, Bulgaria) with the first resonance frequency in the range between 204–497 kHz. The images were captured at a scan rate of 0.5 Hz and on 512 lines. The calibration of the film thickness was obtained by scratching the sample surface with a scalpel in three separated regions. Then step profiles of the three scratches were measured. The procedure was repeated on three different samples made under the same preparation conditions.

The optical images were obtained with a Mitutoyo microscope (Mitutoyo, Kawasaki, Japan), having objectives 10× and 20× and an eyepiece 2×.

## 3. Results and Discussion

### 3.1. Microgels: Preparation and Characterization

**Figure 1:**
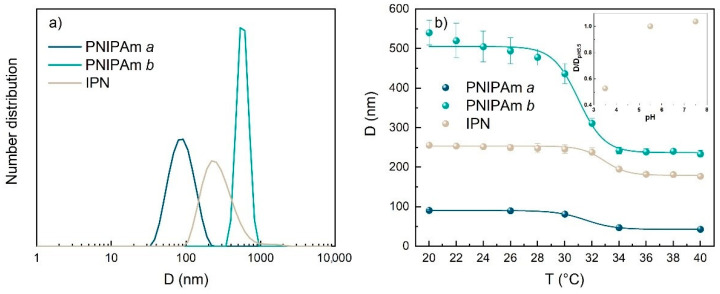
Dynamic light scattering measurements of PNIPAm *a*, PNIPAm *b* and IPN: (**a**) number distributions versus the hydrodynamic diameter measured at 20 °C; (**b**) hydrodynamic diameter versus temperature. In the inset in (**b**), ratio between the diameter at a given pH to the one at pH 5.5 as a function of pH. The concentration of the measured dispersions was 0.01 wt%.

Two PNIPAm microgels, PNIPAm *a* and PNIPAm *b*, with a comparable crosslinking degree but different size, were obtained by copolymerization of NIPAM and BIS in the presence of different surfactant amount. The diameter values measured with dynamic light scattering (DLS) were 91 ± 2 nm and 558 ± 24 nm, for PNIPAM *a* and PMIPAM *b* that were obtained with SDS/NIPAM molar ratio of 5.71 and 0.12, respectively (Table 1 and Figure 1a). In spite to the difference in size the two samples exhibited similar swelling ratio (D_20 °C_/D_40_~2.1–2.2) across the volume phase transition (VPT) that occurred in both samples at around 32 °C. The comparable high swelling ratio can be ascribed to the comparable low crosslinking degree (BIS/NIPAM molar ratio = 0.013) of the two samples.

A microgel sample with a more complex IPN architecture was obtained by copolymerization of AAc and BIS in the presence of PNIPAm *a* microgels [38]. The obtained sample was analyzed with FT-IR spectroscopy and compared with the PNIPAm’s ones (Figure S1). PNIPAm *a* and *b* spectra show all the characteristic peaks of polyacrylamide: 3437 cm^−1^ (N–H and water o–H stretching); 3074 cm^−1^ (acrylic C–H stretching); 2972, 2933, 2875 cm^−1^ (aliphatic C–H stretching); 1642 cm^−1^ (C=O stretching of amide); 1549 cm^−1^ (N–H bending, amide II), 1460 cm^−1^ (symmetric bending  of methyl in  −C(CH_3_)_2_); 1386, 1368 cm^−1^ (asymmetric bending of methyl in  −C(CH_3_)_2_), 1241 cm^−1^, 1175 cm^−1^. As expected, due to the identical composition, no difference was noticed between the two samples that differ only for the particle size. The effective incorporation of Poly(Acrylic Acid), PAAc, into the PNIPAm particles to form the IPN structure, was confirmed with the appearance of a new band in the IR spectrum at 1725 cm^−1^ (Figure S1) that can be attributed to the C=O stretching of carboxylic acid moiety of PAAc.

The amount of the incorporated PAAc in the IPN structure was obtained combing ^1^H-NMR and elemental data [41] (Figure S2) and was estimated in 19.2 wt% (Table 1). The remaining part was composed of PNIPAm 73.6 wt% and BIS 7.2 wt%.

The incorporation of PAAc into PNIPAm *a* to obtain the IPN structure results in a growth of the microgel size (Figure 1a): IPN diameter obtained with DLS was 254 ± 12 nm at 20 °C. The sample underwent a volume phase transition at around 32 °C, as foreseen for PNIPAm-based microgels (Figure 1b). The values of swelling ratio between the diameters at 20 and 40 °C (Table 1) indicate that IPN shrink less than PNIPAm. This is attributable to the presence of the second network in the IPN particles that add a constrain to prevent the collapse of the PNIPAm network. The pH-responsivity of IPN microgel was evaluated by determining the radius of the particles at three different pH: 3.5, 5.5, and 7.5 (inset in Figure 1b). As expected, the PAAc network collapses at pH values below the pKa of AAc (4.5), since the carboxylic acid groups are fully protonated and can form hydrogen bonds with the COOH groups of other AAc repeating units and with the CONH groups of NIPAM units inside the particles. At higher pH, the carboxylic acid groups are partially or fully dissociated and repulsive interactions between the moieties, mentioned above, are established [46]. Thus, a more swollen network with a higher particle diameter can be observed (inset Figure 1b).

### 3.2. Film Characterization

**Figure 2:**
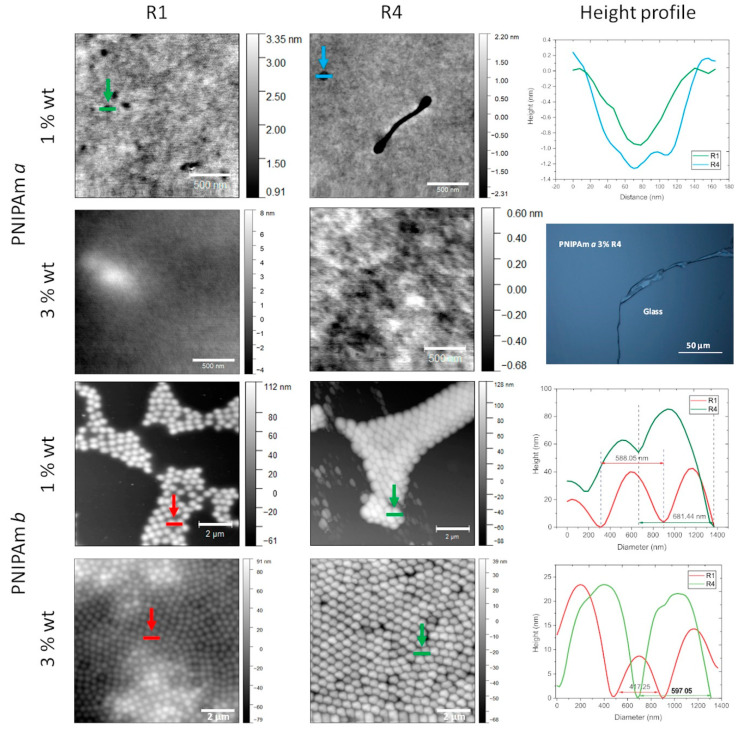
AFM images of PNIPAm *a* (from top, rows 1 and 2) and PNIPAm *b* (rows 3 and 4) films spin-coated at different concentrations (1 wt%, rows 1 and 3; 3 wt%, rows 2 and 4) and different spin-speed values (from left: column 1, R1 = 2000 rmp; column 2, R4 = 5000 rpm). Graphs on column 3, rows 1, 3, and 4, display the height profiles measured in correspondence of the lines shown in the images of column 1 (red lines) and 2 (green). On column 3, row 2, an image of the sample, obtained via optical microscopy.

Firstly, we investigated the conditions under which continuous and uniform films can be obtained. In particular, we varied the dispersion concentration and spin-speed. In Figure 2, we show AFM images obtained for PNIPAm *a* and *b* spin-coated samples. Let us start with PNIPAm *b*, where the effects due to concentration and speed are particularly clear. For a concentration of 1% and speed R1, it is evident that a continuous film does not form but only isolated patches of aggregated particles. This result is in agreement with the ones previously reported by other groups for microgel with a size comparable to or larger than PNIPAm *b* [52,62]. It must be noticed that the spherical shape of the particles can be still observed. However as deduced from the height profiles, they are squeezed down. At the highest speed R4, the coverage is still not continuous but more compact aggregations form with some deformation of the particles, which do not have a spherical shape. For a concentration of 3%, a complete surface coverage can be observed at all speed values. These results suggest that the critical concentration in order to obtain full coverage for this PNIPAm size is between 1% and 3%.

**Figure 3:**
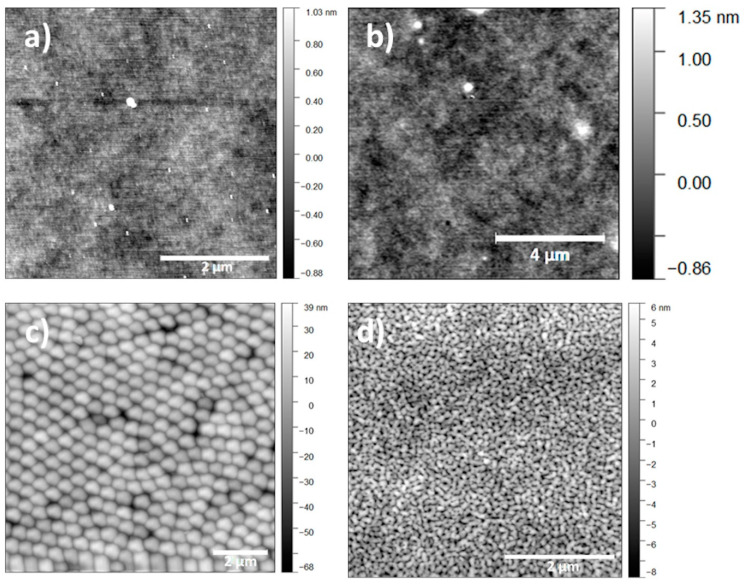
AFM images of (**a**) glass, (**b**) PNIPAm *a*, (**c**) PNIPAm *b*, and (**d**) IPN. Each film spin-coated on a glass-OH surface at a concentration of 3 wt% and speed R4 (5000 rpm).

In summary, PNIPAm *b* exhibits a topography similar to the ones previously reported in the literature for other microgel films [32,33,34,58], whereas PNIPAm *a* presents a topography similar to that observed for linear polymers [67] (Figure 3). This different behavior should be ascribed to the difference in size between the microgels, as this is the only parameter changed. Indeed, it was previously reported that particles with a diameter less than 100 nm and with a low crosslinking density, such as those of PNIPAm’s studied in this work (BIS/NIPAM molar ratio = 0.013), show a behavior more similar to non-crosslinked polymer coils than to hard-sphere colloids. On the contrary, the hard sphere behavior is typical of larger particles, even with a low crosslinking density [65], such as the case of PNIPAm *b* [65,68]. Accordingly, for PNIPAm *a* the ratio between its hydrodynamic (R_H_) and gyration (R_g_) radius was previously reported to be 1.8 [41]. This indicates a low compact structure, more similar to a swollen coil than to a hard sphere, whose theoretical R_g_/R_H_ value is 0.78. Thus, similarly to the chain entanglements occurring during the liquid-to-solid transition of linear polymers, the formation of continuous films for PNIPAm *a* can be attributed to dangling chain ends in the corona of these small particles that interpenetrate considerably upon increasing particle packing [69,70].

Notice that recently, 400–700 nm large and very low crosslinked PNIPAm particles prepared in the absence or with a very low amount of crosslinker BIS/NIPAM molar ratio = 0.005) were reported to be subjected to larger deformation during the absorption on a substrate than particles with a higher crosslinking density (e.g., BIS/NIPAM molar ratio = 0.05) [71,72]. This result may suggest that the particle size at which a change in behavior, from rigid sphere to random coil, may depend on the crosslinking degree. Smooth continuous films can be obtained even with larger PNIPAm particles if their crosslinking degree is relatively low. PNIPAm *b*, which gives a continuous films but with particle edges well detectable, was prepared with an intermediate crosslinker amount (BIS/NIPAM molar ratio = 0.013) with respect to the samples studied by Richtering and Holderer groups [71,72]. In any case, further experiments would be necessary to confirm this hyphotesis.

For IPN, the AFM analysis shows the formation of a flat and continuous film (Figure 3d). The morphology does not depend on the concentration and spin-speed (Figure S3). Only the film obtained for 1% and R4 presents some holes with a depth of about 10 nm. This is probably due to the low thickness and concentration values [73]. The formation of smooth films can be explained in two ways: the interpenetration of the particles is promoted because either the particle diameter or the density are below a critical value. Actually, IPN particles were synthesized by polymerizing a PAAc network inside preformed PNIPAm microgels, so that they have a dense crosslinked core of PNIPAm and PAAc. Furthermore, some of us reported that for a PAAc content larger than 8 wt%, such as the IPN used in the present work, the PAAc that starts growing in the core continues outside the particles forming a loose corona [46]. Thus, the particles can be described as core-shell structures, with a rigid and compact core due to the presence of two interpenetrated networks of PNIPAm and PAAc, and a less dense corona made of only PAAc. Accordingly, IPN microgels with a PAAc content of 19.2%, comparable to that one here studied, were found to exhibit an R_g_/R_H_ ratio value of 1.4, which is intermediate between the one of PNIPAm *a* and the one of a rigid sphere [41]. This value supports the hypothesis of a dense core with the presence of a low density and deformable shell. The low density and deformability of the shells may explain the ability of the particles to form flat films. However, comparing the topography of the IPN and PNIPAm *a* film (Figure 3), we notice that the IPN surface appears to be more irregular and rough than the latter. These differences can be due to the particle structure, as well as to the larger polydispersity of the IPN particles compared to PNIPAm *a*. In any case, the larger size of IPN, compared to PNIPAm *a*, may also contribute to the higher value of the roughness.

Notice that the ionic charge of the particle surface seems to play a minor role in the film formation by spin-coating and in the corresponding morphology. In fact, even though the exact charge of the microgel surface is not known, the estimated values reported in paragraph 3.3 show that the sample for which the particle interpenetrate less, namely PNIPAm *b*, is the one with the lowest surface charge.

**Figure 4:**
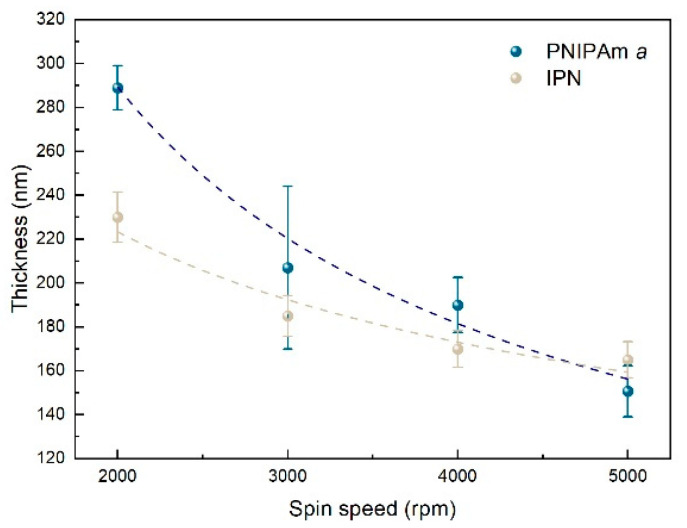
Thickness versus spin-speed for PNIPAm a and IPN films (concentration of 3 wt%) on glass-NaOH.

For microgels forming flat films, namely PNIPAm *a* and IPN, the dependence of the film thickness on the spin-speed was also investigated. The thickness was evaluated as follows: a scratch is made at the centre of the sample and the scratch height can be measured by means of AFM. The average values are found to inversely depend on the spin-speed for both PNIPAm *a* and IPN (Figure 4). At equal spin-speed, the thickness values are larger for PNIPAm *a* than for IPN, except in the case of the largest speed R4, namely 5000 rpm, where comparable values of almost 160 nm are observed for both films. For PNIPAm *a*, the value is larger than the diameter of a single particle in its collapsed state, which is about 40 nm. This suggests that the films are not made of a single layer of particles. For IPN, the diameter in the collapsed state is about 130 nm, making it more complex to state whether it is a monolayer or a multilayer of stretched particles. Thickness versus speed plots can be fitted with a power-law equation, as reported in most experimental works and models [74,75,76,77]: a clear decrease of thickness can be observed when increasing the spin-speed, due to the deformation and interpenetration caused by the ever increasing centripetal acceleration. At R4, the thickness of the two films is about the same. This is probably a consequence of the high speed that allows the interpenetration of the particles, in particular of the less crosslinked shell of IPN, due to the PAAc network. Thus, the thickness is mainly determined by the PNIPAm-core of the particles, equal for both microgels, while the PAAc shell leads to only a slightly higher thickness.

### 3.3. Film Stability

Secondly, we studied the film stability to water soaking. Continuous microgel films were deposited on surfaces that presented different degrees of hydrophilicity. In the literature, it has been widely reported that PNIPAm microgels prepared by precipitation polymerization has a negative charge at their surface when KPS is used as an initiator [46]. These microgels are not stable in water when deposited over neutral glass [59]. On the contrary, they are stable on positively charged surface, usually obtained by PEI deposition, due to the attraction between opposite charges [22,51,52,62,72]. However, to the best of our knowledge, no specific investigation has been reported on the effect of the surface polarity. This should have a different effect on the stability of the films above and below LCST, because of the change in polarity of the film at this temperature.

Washing was thus performed at two different water temperatures (20 °C or 50 °C), below and above LCST. After washing we performed a scratch test, which consisted in cutting the film with a scalpel and observing it with an optical microscope. In the case of hard substrates, such as glass, nothing can be seen while for soft coatings a scratch is visible. Data are reported in Table 2 and the optical observations in Supplementary Materials.

Three kinds of surfaces with decreasing degree of hydrophilicity—named glass-NaOH, glass-TMSPMA, and glass-TMOS—were obtained by treating the cover glasses with NaOH, TMSPMA, and TMOS, respectively. The actual hydrophilicity was evaluated via contact angle measurements and the results are summarized in Table 3. A value of 34.5° was obtained for glass-NaOH, as a consequence of the hydrolysis of the pristine Si–O–Si bridges of glass with the formation of hydroxyl groups at the surface (Scheme 1a) [78]. The surface hydrophilicity after this treatment was lower than after the activation with Piranha solution, which was instead performed before silanization with TSPMA and TMOS. However, both values are in agreement with previous literature data, in particular, if the difference between the specific treatment conditions with NaOH are taken into account [79]. An intermediate hydrophobicity is observed (contact angle value of 70.2°) after silanization with TMSPMA, a moiety that, thanks to the presence of both a carbonyl and of a double carbon–carbon group, has a balanced hydrophilic and hydrophobic character (Scheme 1b). On the other hand, the highest value of 100.6° is measured for glass silanized with TMOS due to the high hydrophobic character of the quite long hydrocarbon chain on the molecule (Scheme 1c).

In spite to the well-known effect of the polarity of the substrate on the film formation by spin-coating, smooth and homogeneous films can be obtained over all the surfaces studied: the AFM analysis evidences the formation of smooth films for PNIPAm *a* and IPN and of a dense layer of particles for PNIPAm *b* on all the substrates (Figure S5).

**Figure 5:**
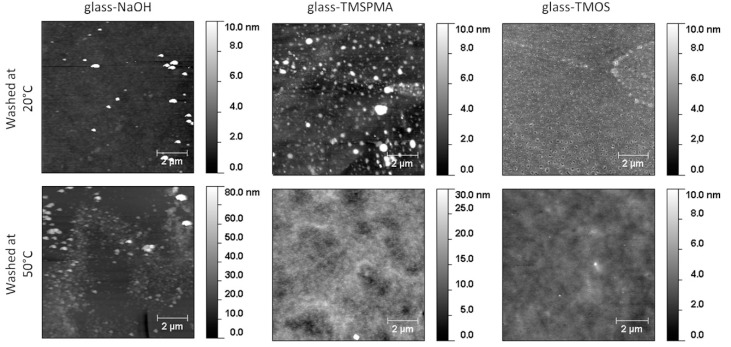
AFM images of PNIPAm *a* films spin-coated on glass-OH, glass-TMSPMA and glass-TMOS at 5000 rpm from a dispersion of 3 wt%, after being washed in water at 20 °C and 50 °C.

After washing the films deposited on glass-NaOH, a scratch can be detected for PNIPAm *a* exposed to water at 20° but not at 50 °C (Figure S6). These results indicate that the film remains attached to the hydrophilic substrate at a temperature when PNIPAm is hydrophilic. On the contrary, at 50 °C PNIPAm behaves as a hydrophobic material leading to the detachment of the film from the polar substrate. In fact, a contact angle of 74.5 ± 0.2° [80] or even higher has been reported for PNIPAm surfaces above the hydrophilic/hydrophobic transition. On glass-TMSPMA, PNIPAm *a* is no longer present after washing at 20 °C, suggesting that the hydrophilic character of the substrate at this temperature is too low with respect to PNIPAm in order to maintain the film attached to the substrate. At 50 °C, instead, the scratch is detected, in agreement with the similar hydrophobic character of the substrate and PNIPAm. The AFM analysis evidences some areas with different thickness and some holes that are not detected in the pristine films, suggesting a partial removal of the film (Figure 5). This observation indicates that the film is stable on the substrate, thanks to the similar interfacial energy. Accordingly, any microgel layer that is not in direct contact with the substrate is not stabilized and is easily washed off. Finally, films deposited on glass-TMOS, with the highest hydrophobicity, are removed at 20 °C but not at 50 °C as for glass-TMSPMA. In this case, the AFM analysis also shows an inhomogeneous surface, rougher than the pristine film, probably due to some removal of the microgel. These results are confirmed by the optical analysis (Figure S6).

**Figure 6:**
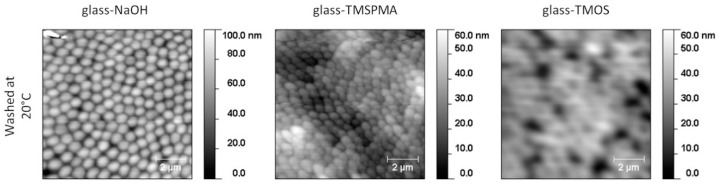
AFM images of PNIPAm *b* films spin-coated on glass-OH, glass-TMSPMA and glass-TMOS at 5000 rpm from a dispersion of 3 wt%, after being washed in water at 20 °C.

After washing at both 20 °C and 50 °C, PNIPAm *b* is still present on all the three different surfaces except on glass-NaOH after washing at 50 °C (Table 2). After washing at 20 °C the morphology seems to evolve in a different way depending on the substrate polarity (Figure 6). In particular, it is not affected on glass-NaOH, where it looks like an array of well distinct particles, and it evolves toward an almost continuous film with the particles that cannot be clearly distinguished in the most hydrophobic substrate (glass-TMOS). These results suggest that below LCST the adhesion between the particles and the substrates is always enough strong to avoid a complete detachment. However, it is not always so strong to prevent partial rearrangement of the particles or detachment at 50 °C. This hypothesis is corroborated by the morphology data obtained after washing at 50 °C, the temperature at which the particles are in their collapsed state and cannot easily rearrange (Figure S4). Comparing PNIPAm *b* with PNIPAm *a* (Table 1), the former seems to be more stable than the latter one, particularly on glass-TMSPMA and glass-TMOS at low temperature. This difference might be attributed to a larger contact area between the substrate and the particles in the case of the bulkier PNIPAm *b* leading to larger adhesion energy that has to be overcome for the detachment of each individual particle.

**Figure 7:**
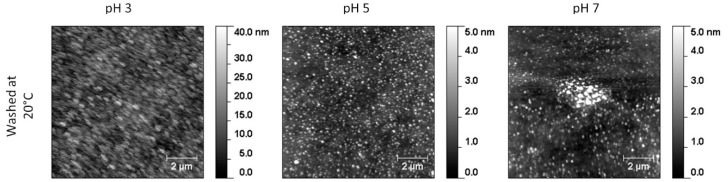
AFM images of IPN films spin-coated on glass-OH, glass-TMSPMA and glass-TMOS at 5000 rpm from a dispersion of 3 wt%, after being washed in water at 50°.

The scratch analysis of IPN films on glass-TMSPMA and glass-TMOS provides the same results as for PNIPAm *a* (Table 2, Figures S8 and S9): the film is present after washing at 50° but not at 20 °C. Moreover, the AFM analysis shows that the film on glass-TMSPMA and glass-TMOS after washing at 50 °C appears no longer continuous, with holes, thus suggesting its partial removal (Figure 7). The hydrophilic state of the particles at 20 °C can be invoked to explain the lack of film stability on these hydrophobic surfaces. On the contrary, the hydrophobicity of PNIPAm above 32 °C provides the stability at 50 °C, thus indicating that PNIPAm keeps its individual surface properties even when incorporated in IPN, to which it provides its peculiar thermo-responsiveness character.

**Figure 8:**
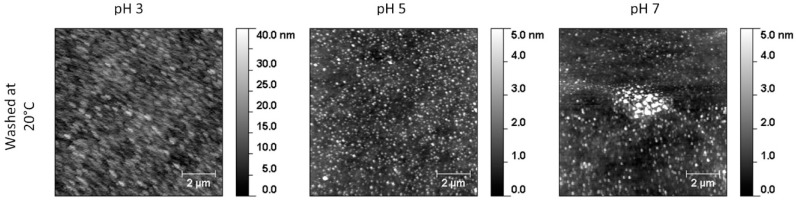
AFM images of IPN films spin-coated on glass-OH at 5000 rpm from a dispersion of 3 wt%, after being washed in water at 20 °C and pH equal to 3, 5, and 7.

Given the presence of PAAc, the pH-responsiveness of IPN was also investigated washing the films with water at a pH value of 3, 5, and 7 (Table 1). The AFM images of IPN films spin-coated on glass-NaOH are reported in Figure 8. The more stable film is the one washed at pH 3, while the ones washed at higher pH values are partially removed or with a more inhomogeneous distribution: an area of few bright particles is visible in the film washed at pH 7. This behavior can be attributed to the different ionization degree and corresponding solubility of the PAAc network. At the lowest pH value, the carboxylic acids are fully protonated and the PAAc chains are not soluble making the particles more rigid and less prone to detach from the surface. At higher pH, they are in a dissociated state, the chains are soluble and the particles bear a negative charge. This gives origin to an electrostatic repulsion between particles that promotes desorption. This result is in good agreement with the one obtained in a previous work where a P(NIPAM-*co*-AAc) microgel was spin-coated on a PEI-modified silicon substrate; the densely-packed particles were stable when immersed in water at a pH of 2 but they desorbed at higher pH values [59].

For IPN, several factors need to be taken into account: the presence of the PAAc network, the intermediate particle size, the deformability and the ionization degree of the AAc repeating unit.

The exact estimation of the ionic charge on the surface of soft microgels is not trivial and is beyond the scope of the present paper. However, in order to study the possible effect of the ionic charge on the stability of microgel films a rough estimation of the particle charge, *Q_particle_*, was performed using Equation (1).

(1) $$Q_{particle}=\frac{Ions\cdot e\cdot N_{A}}{\frac{\varphi \cdot V_{tot}}{\frac{4}{3}\pi R^{3}}}$$

where *Ions* is the amount of ionizable groups in the sample, *e* is the charge of an electron, *N_A_* is the Avogadro constant, φ is the volume fraction of the particles, *V_tot_* is the total volume of the solution, and *R* is the hydrodynamic radius of the particles as obtained by DLS measurements (Table 1). In the case of PNIPAm microgels prepared with KPS initiator [46,81], *Ions* is calculated as

(2) Ions (PNIPAm)=2 [I]

where [*I*] is the number by mole of initiator (KPS), and 2 takes into account the charge number for each moiety. In the case of IPN, in addition to the initiator moieties, the ionic charge can be due to ionized carboxylic acid groups as well and *Ions* is calculated as

(3) Ions (IPN)=2 [I]+f[AAc]

where [*I*] is the total amount of initiators used in the first and second reaction steps (KPS and NH_4_PS), AAc is the number per mole of carboxylic acid groups and *f* is fraction amount of ionized carboxylic acid group. *f* depends on pH and was assumed to be the same as for pure PAAc [82].

The volume fraction values, φ, used for the calculation, are 0.775 for PNIPAm *a* [83], 21.11 for PNIPAm *b* [84], and 0.098 for IPN (at pH 5.5 [83]), as obtained through rheological measurements. Notice that in the case of PNIPAm *b*, for which an exact value was not available, a preliminary estimated value was used, which is comparable with results obtained from other researcher with similar microgels [85].

The surface charge density of the particle surface (Table 4) is thus calculated assuming that all ions in each microgel particle are located at the surface or close to it

(4) $$Q_{surface}=\frac{Q_{particle}}{4\pi R^{2}}$$

This assumption is widely accepted for PNIPAm microgels [86], where the ionizable groups (the sulfate moieties) are located at the chain-end. In fact, the most mobile chain-ends usually prefer to locate at the surface. In the case of IPN, the localization of the ionized groups has not been studied yet. However, most likely, the carboxylic acid groups in the internal part of the particles are less ionized and have been involved in multiple hydrogen bonds with an amide functionality. This is particularly true at pH values below 7, where only one over three groups is ionized [82]. Therefore, ionized groups can be reasonably assumed to be located on a thin shell close to the surface, also in the case of IPN.

The surface charge values obtained under the above described assumption (Table 4) is negative for all samples, in agreement with mobility values previously reported for similar samples [46]. By comparing the values above and below LCST, the former appears larger than the latter due to the contraction of the particle size, with a corresponding concentration of the charge for surface unit. In the case of PNIPAm *b* and IPN, the density becomes roughly double, while in the case of PNIPAm *a* it may increase to three times. However, this difference seems to have no apparent correlation with the stability on the substrate. Instead, the net density of charge seems to play a major role, in particular below LCST/VPT where microgels are in their swollen state. Under this condition, PNIPAm *b*, having the lowest surface charge density, seems to be the most difficult to wash off. In this case, the reduced repulsion between the microgel particles should play a role in avoiding the detachment of the particles from the substrate.

As expected, IPN particles bear a surface charge at least 10 times larger than PNIPAm ones, both below and above VPT, due to the presence of AAc moieties. Accordingly, at the pH of 5.5, at which the surface charge was calculated, the film is washed off from all the substrates. The film is stable only at pH 3. At this pH the surface charge could not be calculated because the exact value of the particle volume fraction is not available. However, even though the particle size and the surface area is smaller than at pH 5.5 (see inset Figure 1b), the ionization degree should be almost zero [82], based on the data of pure PAAc. Then a surface charge close to the values obtained for PNIPAm *a* and *b* is expected with a comparable stability, at least on the polar glass-NaOH surface.

The comparison between the stability of PNIPAm *a, b* suggests that in addition to the surface charge effect, the film morphology and the particle deformability, that in turn depends on the PNIPAm properties, may also play a role in the film stability. The addition of PAAc, containing carboxylic acid groups, adds extra responsiveness to the environment and allows one to tune the hydrophilicity of the whole system through a protonation/deprotonation equilibrium. In fact, while in the case of PNIPAm the net surface charge is very low, this is not true in the case of IPN due to the possible dissociation of the carboxylic acid group.

IPN is more hydrophilic than PNIPAm at neutral and basic pH, where the carboxylic groups are deprotonated and bear a negative charge, but it is less hydrophilic at acidic pH, where all the groups are in the protonated form. This extra parameter acting on the hydrophobicity of the system via pH also allows one to control the film stability.

## 4. Conclusions

Thin microgel films, made of PNIPAm or PNIPAm-PAAc interpenetrated polymer network, have been spin-coated on glass surfaces with various degrees of hydrophilicity. Continuous and flat films, with a thickness dependent on the dispersion concentration and spin-speed, have been obtained for PNIPAm and IPN particles having a diameter of 91 and 254 nm, respectively. For PNIPAm particles with a diameter of 558 nm, dense arrays of distinct particles have been instead observed.

The stability of the continuous films, upon washing in water, is found to depend on the hydrophilic matching between the substrate and the microgel composition. Whereas the hydrophilicity of the films depends on the temperature thanks to PNIPAm. We have also observed that the presence of PAAc inside the IPN particles can change the microgel behavior. Due to the presence of a charge inside the particles, pH can also affect the film morphology. Furthermore, in the case of a hydrophilic surface, such as glass treated with NaOH, the ionic charge of the particle surface is the key parameter controlling the film stability.

Therefore, we conclude that, with a proper selection of the surface hydrophilicity and the environmental conditions, it is possible to manipulate the film morphology as well as its stability thanks to the stimuli-responsiveness of the microgels.

The film stability on the surface under different conditions has a direct implication on their use in the fabrication of devices for biomedical applications. For instance, surfaces with patterned modifications can allow selectively removing the gel film from some specific area exploiting the different stability under water washing conditions.

## Figures and Tables

**Table 1:** Microgels physical-chemical characteristics

	Diameter (nm) at 20 °C	Swelling Ratio D_20 °C_/D_40 °C_	BIS* (wt%)	PAAc* (wt%)
**PNIPAm *a***	91 ± 2	2.1	1.4%	0%
**PNIPAm *b***	558 ± 24	2.2	1.3%	0%
**IPN**	254 ± 12	1.3	4.9%	19.2%

* Percentage amount in the purified microgel.

**Table 2:** Film stability evaluated, after water washing, with a scratch test. Yes = the film was still detectable after washing; No = the film could not be detected after washing.

Substrate	Coating Stability							
	PNIPAm *a* Wash 20 °C	PNIPAm *a* Wash 50 °C	PNIPAm *b* Wash 20 °C	PNIPAm *b* Wash 50 °C	IPN wash 20 °C			IPN Wash 50 °C
glass-NaOH	Yes	No	Yes	No	pH3 Yes	pH5 No	pH7 No	No
glass-TMSPMA	No	Yes	Yes	Yes	No			Yes
glass-TMOS	No	Yes	Yes	Yes	No			Yes

**Table 3:** Contact angle values of pristine and functionalized glasses. Each value is an average of three measurements.

Surface	Contact Angle
Glass	47.3 ± 0.7
Glass treated with Piranha	<10°
glass-NaOH	34.5 ± 1.9
glass-TMSPMA	70.2 ± 4.1
glass-TMOS	100.6 ± 0.9

**Table 4:** Charge density at the microgel surface calculated at 25 °C and 38 °C

	*Q_surface_* × 10^20^ (C/nm^2^) 25 °C	*Q_surface_* × 10^20^ (C/nm^2^) 38 °C
PNIPAm *a*	−0.74	−2.1
PNIPAm *b*	−0.18	−0.36
IPN at pH 5.5	−13	−24

## Supplementary Materials

The following are available online at https://www.mdpi.com/2073-4360/12/11/2638/s1, Figure S1: ATR FT-IR spectra of PNIPAm *a*, PNIPAm *b* and IPN; Figure S2: ^1^H-NMR spectra of PNIPAm *a* and IPN microgels in D_2_O; Figure S3: AFM images of IPN films prepared under different spin-speed values and dispersion concentrations on glass-NaOH; Figure S4: AFM images of PNIPAm *b* films on different substrates after washing at 50 °C; Figure S5: AFM images of PNIPAm *a*, PNIPAm *b* and IPN films on substrates with different degrees of hydrophilicity; Figure S6: Optical images of scratches made on washed PNIPAm *a* films; Figure S7: Optical images of scratches made on washed PNIPAm *b* films; Figure S8: Optical images of scratches made on IPN films washed at 20 °C; Figure S9: Optical images of scratches made on IPN films washed at 50 °C.
