# Supplementary material for: Formation and Stability of Smooth Thin Films with Soft Microgels Made of Poly(N-Isopropylacrylamide) and Poly(Acrylic Acid)
Source: Polymers (Basel). 2020 Nov 10;12(11):2638. doi: 10.3390/polym12112638 (PMC7697199; doi:10.3390/polym12112638)
Supplement: Supplementary file 1 [file polymers-12-02638-s001.pdf]

Supplementary Materials

Formation and stability of smooth thin films with soft microgels made of poly(N-isopropylacrylamide) and poly(acrylic acid)

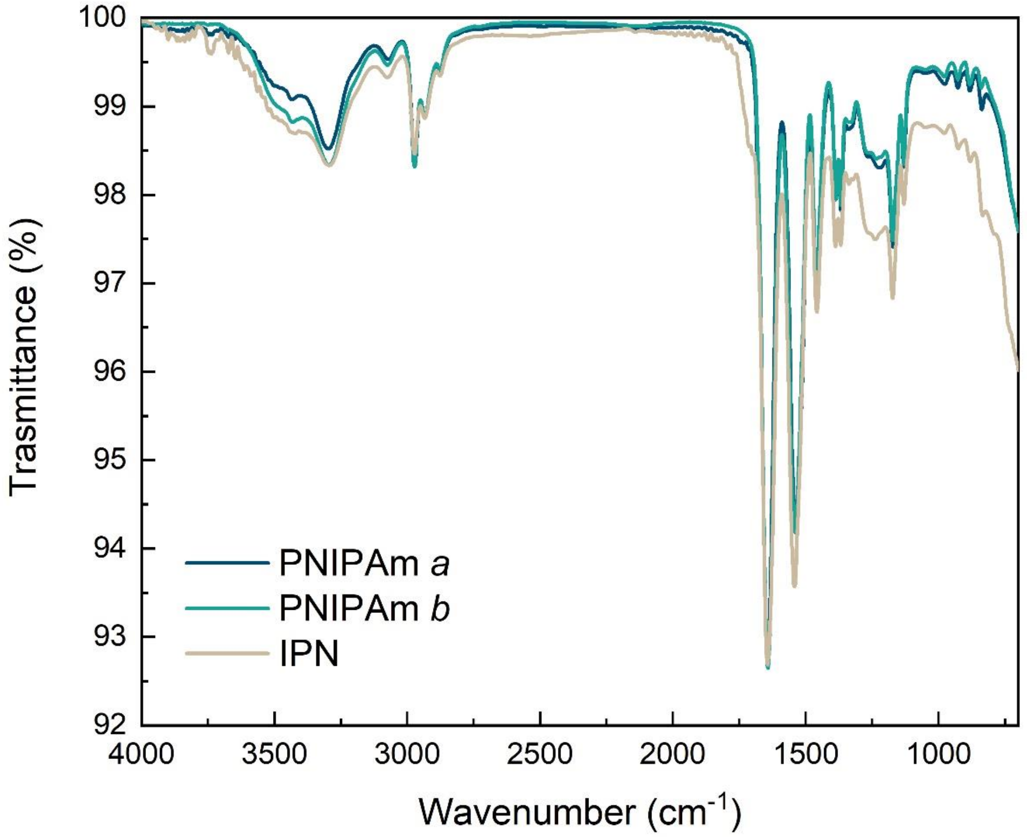

Figure S1. ATR FT-IR spectra of PNIPAM *a*, PNIPAM *b* and IPN. The spectra are normalized with respect to the adsorption band at 1645 cm^{-1}, due to the PNIPAm network. Characteristic peaks of PNIPAM microgels: 3437 cm^{-1} (N-H and water O-H stretching), 3074 cm^{-1} (acrylic C-H stretching), 2972, 2933, 2875 cm^{-1} (aliphatic C-H stretching), 1642 cm^{-1} (C=O stretching of amide), 1549 cm^{-1} (N-H bending, amide II), 1460 cm^{-1} (symmetric bending of methyl in -$C(CH_3)_2$), 1386, 1368 cm^{-1} (asymmetric bending of methyl in -$C(CH_3)_2$), 1261, 1172 cm^{-1}. An additional peak at 1725 cm^{-1} in the IPN spectrum can be attributed to the C=O stretching of carboxylic acid moiety of PAAc. The presence of this peak proves the presence of the PAAc network in addition to the PNIPAM one.

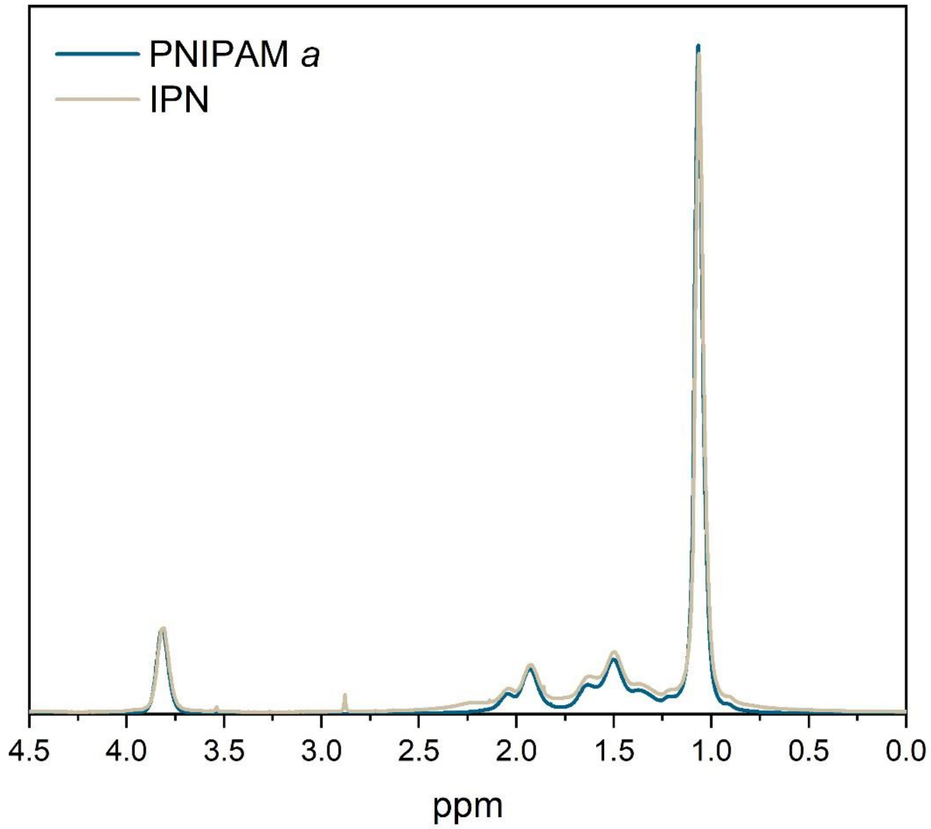

Figure S2. ^{1}H-NMR spectra of PNIPAM *a* and IPN microgels in D_2O. The chemical shift of peaks for all the samples is reported in the following.

PNIPAm *a* (401 MHz, D_2O) δ = 3.81, 2.04, 1.93, 1.82, 1.63, 1.50, 1.38, 1.22, 1.07, 0.93.

PNIPAm *b* (401 MHz, D_2O) δ = 3.81, 2.04, 1.92, 1.82, 1.63, 1.49, 1.36, 1.22, 1.06, 0.90.

IPN (401 MHz, D_2O) δ = 3.81, 2.88, 2.04, 1.93, 1.86, 1.63, 1.50, 1.36, 1.06, 0.91.

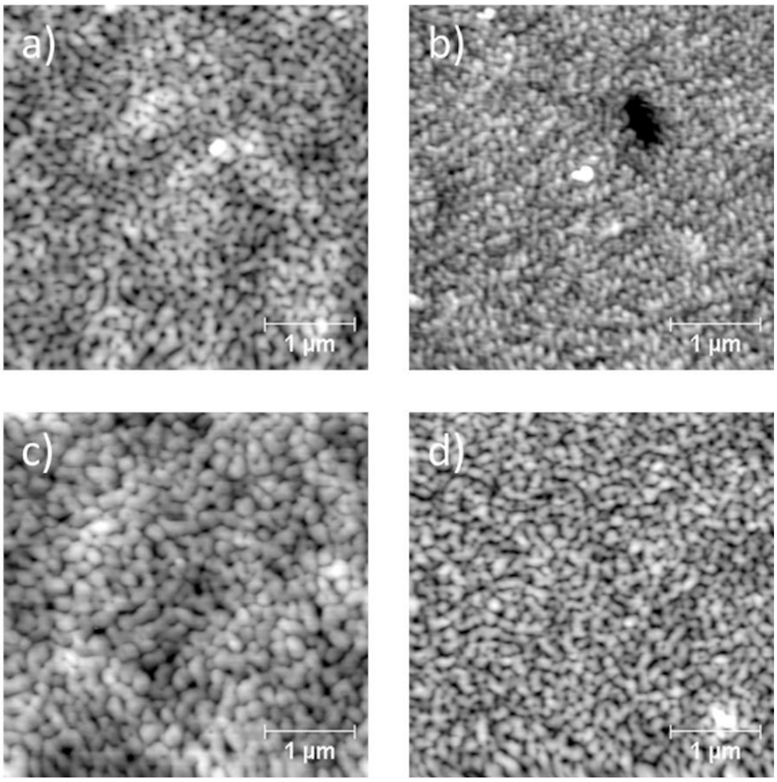

Figure S3. AFM images of IPN films obtained from spin-coating dispersions with the following concentration and spin speed values: a) 1% and 2000 rpm , b) 1% and 5000 rpm, c) 3% and 2000 rpm, d) 3% and 5000 rpm.

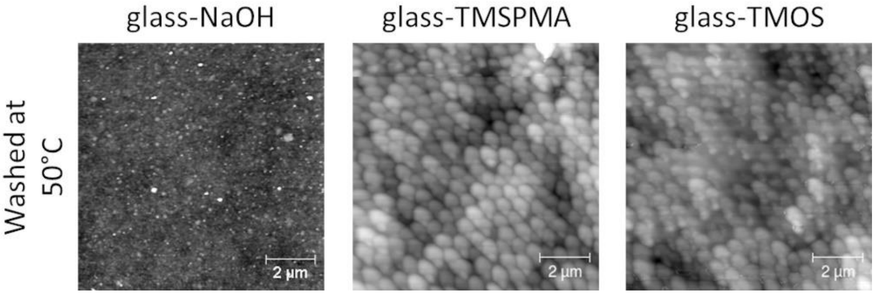

Figure S4. AFM images of PNIPAM *b* films spin-coated on (a) glass-OH, (b) glass-TMSPMA and (c) glass-TMOS, washed at 50 °C.

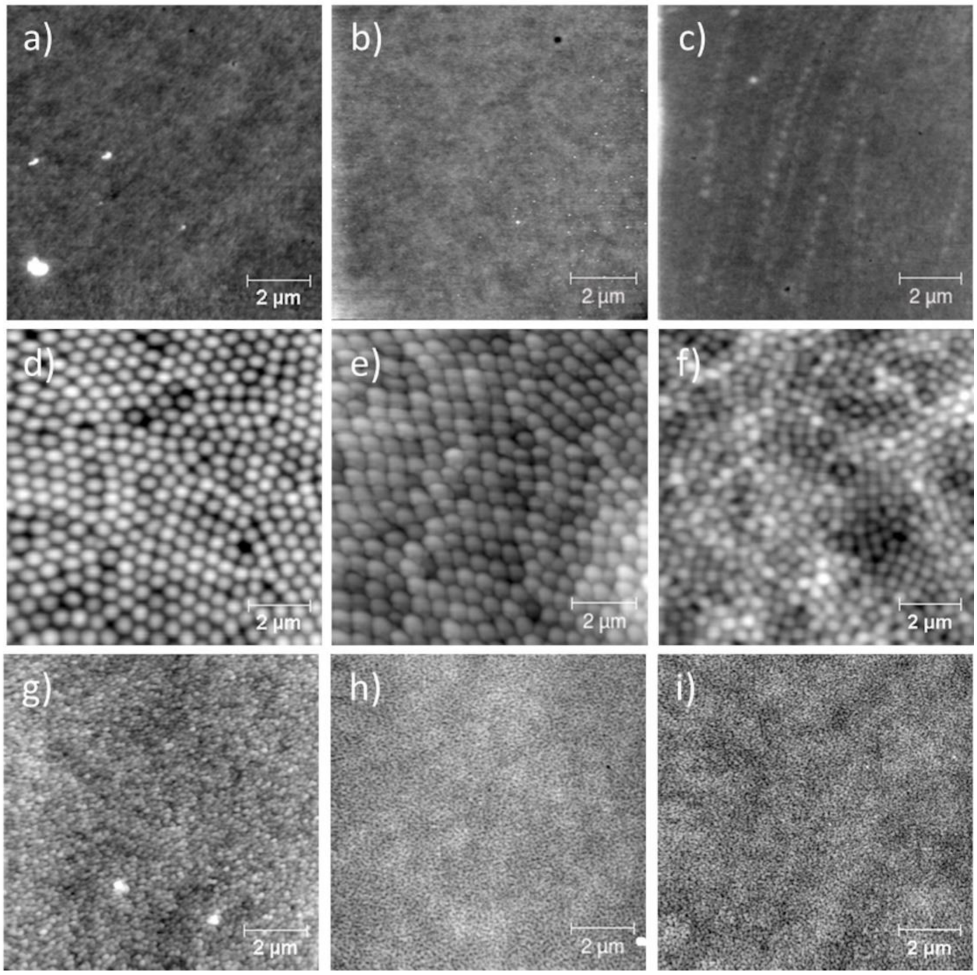

Figure S5. AFM images of microgels spin-coated on glass treated surfaces. PNIPAM *a* on (a) glass-NaOH, (b) glass-TMSPMA, (c) glass-TMOS. PNIPAM *b* on (d) glass-NaOH, (e) glass-TMSPMA, (f) glass-TMOS; IPN on (g) glass-NaOH, (h) glass-TMSPMA, (i) glass-TMOS.

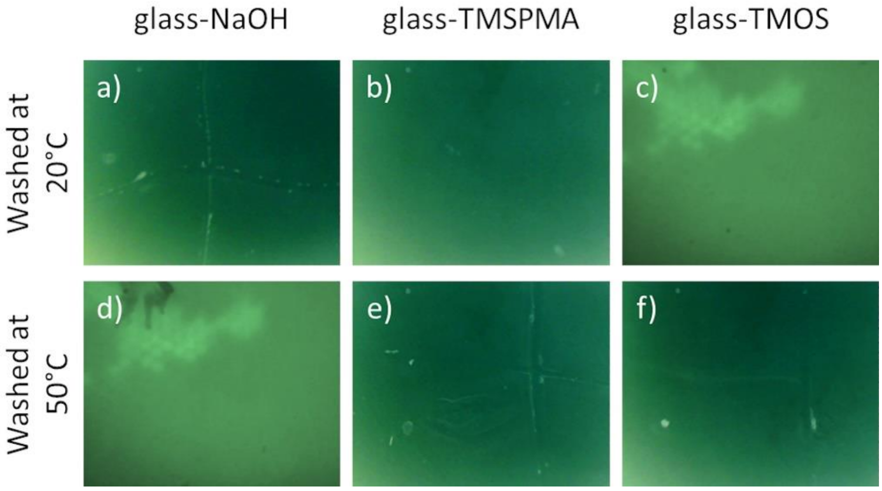

Figure S6. Optical images of scratches made on washed PNIPAM *a* films. The scratches are visible in panel (a), (e) and (f).

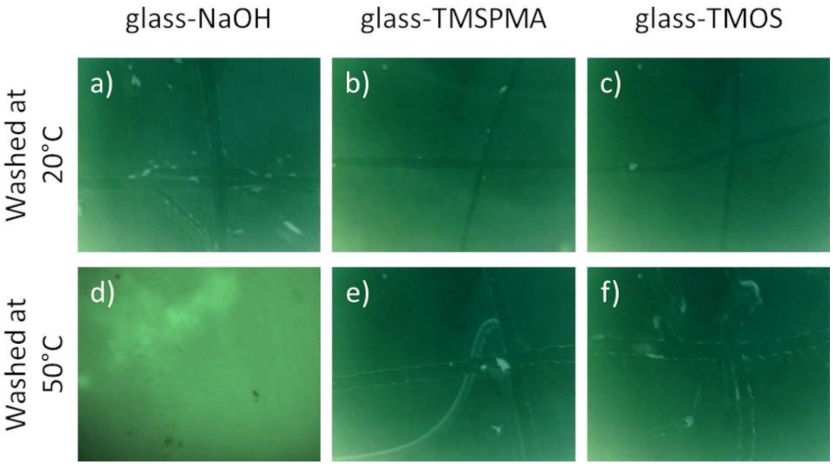

Figure S7. Optical images of scratches made on washed PNIPAM *b* films. The scratches are visible in panel (a), (b), (c), (e) and (f).

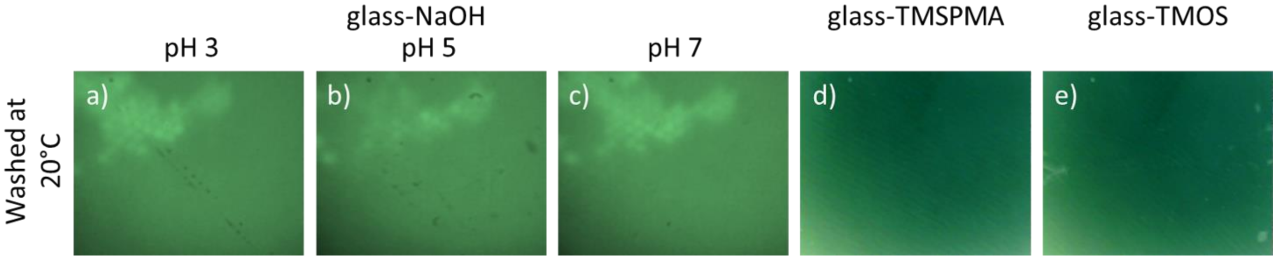

Figure S8. Optical images of scratches made on IPN films washed at 20°C.

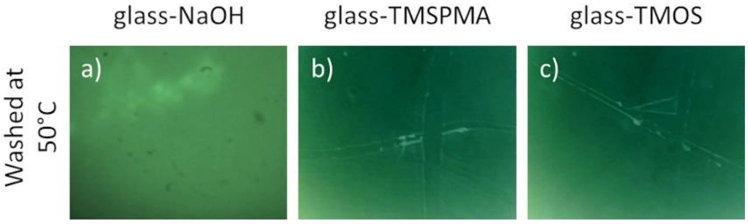

Figure S9. Optical images of scratches made on IPN films washed at 50°C.
